# A Rare Case of Isolated Congenital Facial Nerve Aplasia in an Infant

**DOI:** 10.7759/cureus.54500

**Published:** 2024-02-19

**Authors:** Shiji Chalipat, Sanjay Chavan, Shailaja Mane, Nikhil Taneja, Gaurav Kumar

**Affiliations:** 1 Pediatrics, Dr. D.Y. Patil Medical College, Hospital & Research Center, Dr. D.Y. Patil Vidyapeeth (Deemed to be University), Pune, IND

**Keywords:** lower motor neuron facial palsy, lower motor neuron lesion, congenital facial nerve palsy, lower motor facial palsy, facial nerve aplasia

## Abstract

Facial nerve aplasia is an exceptionally rare condition, with only a few reported cases in the existing medical literature. Congenital facial palsy (CFP) is characterized by the clinical manifestation of facial paralysis involving the seventh cranial nerve, either evident at birth or shortly thereafter. This condition is categorized based on various parameters, including the presence of trauma or developmental origins, unilateral or bilateral involvement, and whether the paralysis is complete or incomplete. While CFP is uncommon, its occurrence can pose multiple challenges for newborns, such as difficulties in nursing and incomplete closure of the affected eye. In cases where the paralysis persists, there is the potential for a long-term impact on the child's speech, emotional expressions, and mastication.

Here we present the case of a six-month-old male child who experienced lower motor neuron palsy of the facial nerve on the left side since birth. This case contributes to the limited knowledge surrounding facial nerve aplasia and CFP, emphasizing the importance of early diagnosis and intervention to mitigate potential long-term complications.

## Introduction

Congenital facial palsy (CFP) is a rare condition, with an incidence estimated to be between 0.8 and 2.3 per 1,000 live births [1]. The origins of this condition are typically attributed to either developmental or traumatic (acquired) causes. Birth injuries, constituting a traumatic etiology, are the most common contributors, often resulting in temporary paralysis that resolves within a few weeks [2]. However, in cases where facial nerve palsy is congenital and associated with facial nerve aplasia, the paralysis tends to be irreversible. Therefore, establishing the etiological diagnosis becomes crucial not only for prognostic considerations but also for medicolegal implications. In the presented case, we report an instance of CFP attributed to facial nerve aplasia, a diagnosis confirmed through neuroimaging techniques. This case underscores the importance of advanced diagnostic tools in identifying the structural abnormalities associated with CFP, aiding in accurate and appropriate medical management.

## Case presentation

A six-month-old male child presented to the pediatric neurology outpatient department exhibiting a consistent deviation of the angle of the mouth to the right side during crying and an inability to fully close the left eye, both of which were observed immediately after birth and remained non-progressive. The child was delivered at term through an uneventful vaginal delivery following an uncomplicated antepartum period, without the need for resuscitation. The absence of instrumentation during delivery and a lack of birth trauma were noted. The APGAR (appearance, pulse, grimace, activity, and respiration) score at birth was within normal ranges, and the birth weight measured 2,550 g.

Parental observation of facial asymmetry, particularly noticeable during crying, was reported from the first day of the child's life. The family history did not reveal any significant instances of inheritable diseases or neurological illnesses. Upon clinical examination, there were no evident dysmorphic features, and neurocutaneous markers were notably absent. Anthropometric measurements, including head circumference, weight, and length, were within normal ranges for the child's age, suggesting that overall growth and development were proceeding normally.

**Figure 1:**
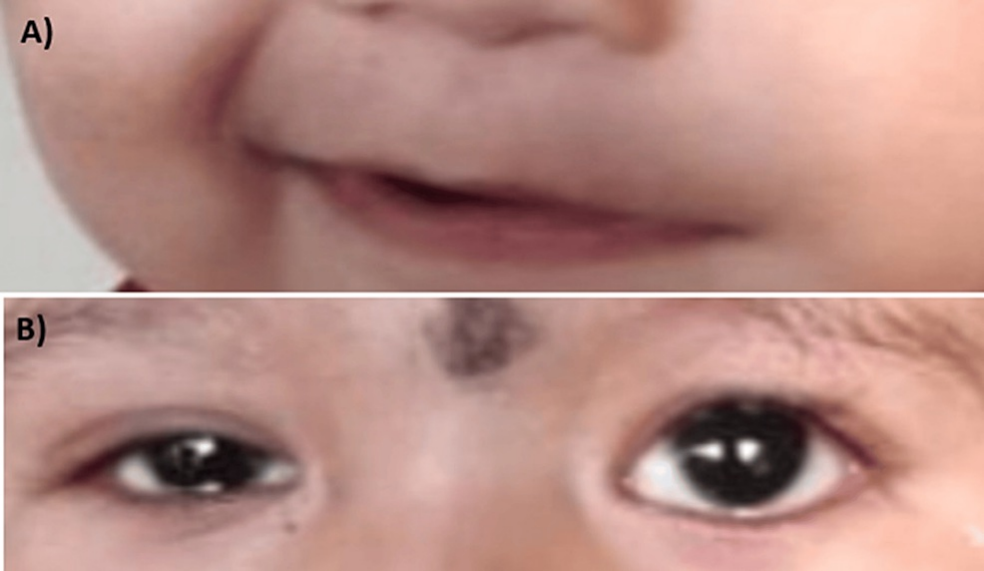
Clinical image of the patient Left lower motor neuron-type facial palsy is evident from a deviation of the angle of the mouth towards the right side while smiling (A) and the inability to close the left eye completely (B).

Cranial nerve examination specifically identified a left lower motor neuron-type facial palsy, characterized by the deviation of the mouth angle towards the right side, particularly pronounced during crying (Figure 1A), and the inability to fully close the left eye (Figure 1B). There were no other observed cranial nerve palsies, and the remainder of the neurological examination yielded normal results. Apart from the left facial paralysis, the child was in overall good health.

**Figure 2:**
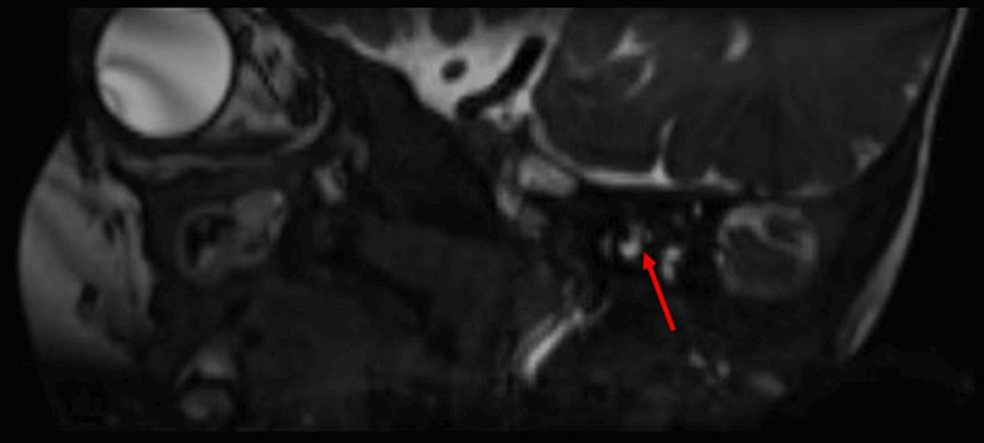
Sagittal sections of the CISS sequence of the MRI of the internal auditory canal on the left side reveal a normal eighth nerve with the absence of the seventh nerve. CISS: constructive interference in steady state

**Figure 3:**
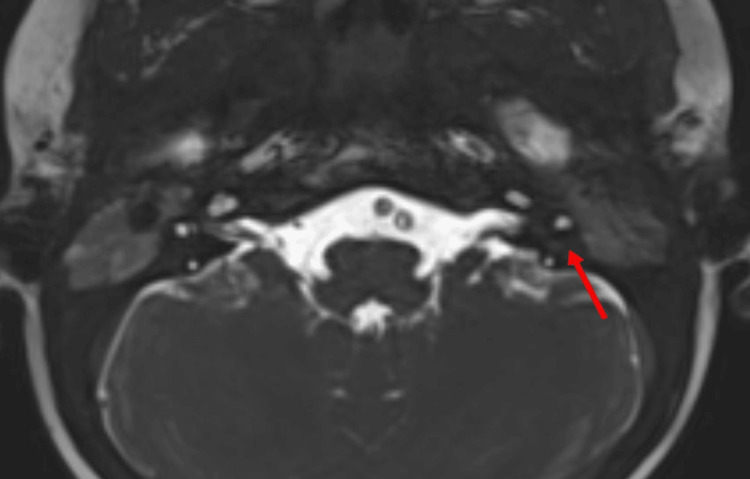
Axial section of the CISS sequence of the MRI of the internal auditory canal It reveals a normal seventh/eighth nerve complex entering the porus acusticus on the right side and the eighth nerve entering the left porus acusticus on the left side. There is aplasia of the seventh nerve on the left side. CISS: constructive interference in steady state

**Figure 4:**
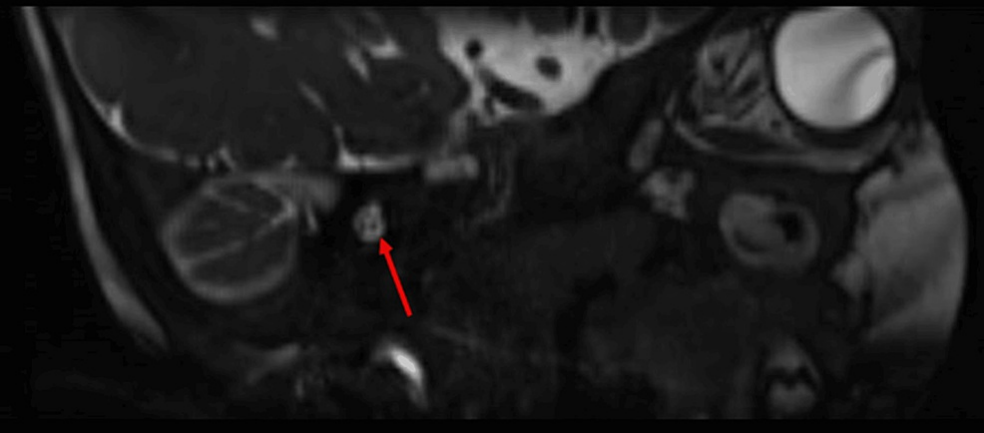
Sagittal sections of the CISS sequence of the MRI of the internal auditory canal on the right side reveal a normal seventh/eighth nerve complex. CISS: constructive interference in steady state

Three-dimensional (3D) constructive interference in steady state (CISS) imaging provided valuable insights into the anatomical details of the patient’s internal auditory canal. The imaging revealed the absence of the left facial nerve in the antero-superior quadrant (Figure 2), while the cochlear nerve was observed in the antero-inferior quadrant (Figure 3). Both vestibular divisions were visualized in the postero-superior and postero-inferior quadrants. These imaging results strongly suggested aplasia of the left facial nerve, contributing to the observed left lower motor neuron-type facial palsy. Sagittal sections of the CISS sequence of the MRI internal auditory canal on the left side further showed the absence of the seventh nerve (facial nerve) while showing a normal eighth nerve (cochlear and vestibular nerves) (Figure 4). This imaging evidence substantiated the clinical findings and aided in establishing a precise diagnosis of facial nerve aplasia, contributing to a comprehensive understanding of CFP in this particular case.

Notably, echocardiography did not reveal any cardiac abnormalities, and brainstem auditory evoked responses were within the normal range.

## Discussion

Congenital facial palsy is a rare condition, primarily attributed to traumatic causes and rarely to developmental anomalies [2]. The facial nerve, with its relatively superior extracranial course, is particularly vulnerable to injury, and the most common etiological factors for CFP include intrauterine posture, perinatal trauma, and intrapartum compression [3].

Developmental causes leading to aplasia or hypoplasia of the facial nerve or nuclei typically manifest with associated cranial involvement. Syndromic associations include Poland syndrome, characterized by CFP accompanied by pectoralis major muscle being absent and upper limb defects; Goldenhar syndrome, encompassing unilateral facial hypoplasia, epibulbar dermoid, cervical vertebral defects, and preauricular skin tags; Moebius syndrome, involving variable degrees of agenesis or hypoplasia of the sixth and seventh cranial nerves or nuclei, usually bilaterally; CHARGE syndrome, involving coloboma, choanal atresia, heart defects, genital abnormalities, retarded growth, and ear malformation; and cardio-facial syndrome, presenting with weakness of the facial muscles [4]. Isolated facial nerve agenesis, or hypoplasia, is rarely reported, with Jervis et al. (2001) documenting the first case of facial nerve agenesis [5].

Our patient had no significant antenatal or perinatal history, and there was no noteworthy information regarding the birth process. Left-sided facial palsy was observed immediately after birth during crying. The child did not experience feeding difficulties and underwent regular follow-ups, with no significant family history noted.

Facial palsy has been associated with anomalies in the pinna and the external auditory canal, ranging from minor abnormalities to more severe conditions such as microtia and atresia. A study conducted by Kondev et al. (2004) highlighted a familial association of unilateral facial palsy in three generations of males within a family, diagnosed through neuroimaging and electromyography. Clinical examination revealed no involvement of other cranial nerves, and a thorough evaluation for syndromic associations was conducted [6].

Given no recovery over time, neuroimaging through an MRI of the brain with a 3D-CISS sequence revealed agenesis of the left facial nerve. The analysis of other cranial nerves, including the abducens, trigeminal, right facial, and cochlear nerves, showed no abnormalities. An MRI, particularly with high-resolution T2-weighted 3D sequences like 3D-CISS, serves as the optimal diagnostic modality for studying the canalicular and cisternal segments of the facial nerve. Thinning or absence of the facial nerve within the canal confirms the diagnosis of hypoplasia or agenesis.

Differentiating between traumatic and developmental causes of CFP is crucial due to its prognostic and medicolegal implications. Most cases of CFP resulting from birth trauma exhibit recovery within a few months, unless there is a complete nerve transaction. Conversely, CFP due to developmental causes is often irreversible and has a poor recovery prognosis. However, Verzij et al., in their Moebius syndrome series, reported mild improvement in facial function or residual activity in some facial muscles [7].

A detailed examination of MRI images, along with an understanding of facial nerve aplasia, provides valuable guidance to radiologists and neurologists in diagnosing this unusual condition. This, in turn, helps in accurate and timely therapeutic decisions and prognoses.

## Conclusions

Congenital facial palsy, due to facial nerve hypoplasia or agenesis, is an extremely rare condition with poor recovery. It can be isolated or associated with other malformative syndromes such as Poland syndrome, Goldenhar syndrome, Moebius syndrome, CHARGE syndrome, Melkersson-Rosenthal syndrome, Ramsay Hunt syndrome, branchio-oto-renal (BOR) syndrome, Brown-Vialetto-Van Laere (BVVL) syndrome, and Miller Fisher syndrome. An MRI of the brain with high-resolution 3D-CISS sequences is the best diagnostic tool to enable the diagnosis. It is imperative to differentiate between developmental and traumatic causes, which will help in accurate and timely therapeutic decisions and prognoses.
